# Euglycemic Diabetic Ketoacidosis and Its Prevention in Elective Surgical Patients Taking Sodium-Glucose Linked Transporter 2 Inhibitors: An International Perspective

**DOI:** 10.1016/j.artd.2025.101840

**Published:** 2025-09-16

**Authors:** James H.J. Selbie, Shuhei Hiyama, Hemant Pandit

**Affiliations:** a Leeds General Infirmary, Leeds, England; b Department of Orthopedics, Jichi Medical University, Shimotsuke, Japan; c Leeds Institute of Rheumatic and Musculoskeletal Medicine (LIRMM), University of Leeds, Leeds, England

**Keywords:** Adult, Perioperative care, Anesthesia, Blood glucose, Diabetes mellitus, Surgery, Humans, Hypoglycaemic agents, Euglycemic diabetic ketoacidosis, Sodium-glucose transporter 2 inhibitors

## Abstract

**Background:**

Sodium-glucose linked transporter 2 (SGLT-2) inhibitors are becoming ubiquitous in medical practice. While beneficial in many areas, they have been implicated in a number of cases of euglycemic diabetic ketoacidosis, a serious and potentially fatal complication, in surgical patients. Therefore, it is important for health professionals to have clear guidelines on how to avoid this. The purpose of this study was to collate and evaluate the available guidelines for the perioperative management of patients taking SGLT-2 inhibitors and to outline the pathophysiology of EDKA in surgical patients.

**Methods:**

A review of the available guidelines was performed using databases from 2010 to 2024. Nine guidelines from across the world were identified and reviewed for specific recommendations related to preoperative withholding time, ketone monitoring, postoperative reintroduction of SGLT-2 inhibitors, and emergency surgery.

**Results:**

The most commonly recommended preoperative withholding time was 4 days for ertugliflozin and 3 days for all other SGLT-2 inhibitors. Most guidelines recommended regular ketone monitoring, but only one presented a detailed strategy. Most guidelines had no recommendation on reintroduction of SGLT-2 inhibitors, but those that did suggested that this should only happen given normal serum ketones and oral intake. Most guidelines had no consideration for emergency surgery, but those that did advocated for immediate treatment cessation.

**Conclusions:**

There was little consensus between the guidelines, suggesting that this is a poorly understood subject. There is clearly a need for dissemination of the pathophysiological basis for the correct management of surgical patients taking SGLT-2 inhibitors, to avoid EDKA.

## Introduction

Sodium-glucose linked transporter 2 (SGLT-2) is a protein channel that exists in the proximal convoluted tubules of the kidneys. It normally functions to actively transport glucose, along with sodium, from the urine back into the blood. SGLT-2 inhibitors block these channels, preventing the reabsorption of glucose, thus reducing blood glucose and improving glycemic control. This is helpful in patients with type 2 diabetes, and the use of these medications alongside insulin is supported by multiple guidelines [1].

The different SGLT-2 inhibitors, their half-lives, and the regions in which they are approved for use are listed in Table 1.

**Table 1:** Summary of the available SGLT-2 inhibitors, their half-lives, and regions in which they are approved for use.

Drug name (generic)	Half-life	Approved regions
Ipragliflozin	15 h [2,3]	Asia
Dapagliflozin	13 h [4]	Europe, North America, Asia, Oceania
Luseogliflozin	14 h [5]	Asia
Tofogliflozin	6 h [6]	Asia
Canagliflozin	11 h [4]	Europe, North America, Asia, Oceania
Empagliflozin	12 h [4]	Europe, North America, Asia, Oceania
Ertugliflozin	17 h [4]	Europe, North America, Oceania
Sotogliflozin	20 h [7]	North America
Bexagliflozin	12 h [7]	North America

The primary clinical indication for SGLT-2 inhibitors is type 2 diabetes. However, they are increasingly used in patients with heart failure and chronic kidney disease. SGLT-2 inhibitors help patients with heart failure by reducing cardiac remodeling through increased diuresis and consequent reduction in preload, as well as by reducing endothelial dysfunction which contributes to a decrease in vasoconstriction and therefore afterload. They help patients with chronic kidney disease by increasing naturesis, which increases erythropoietin production through increased tone in the afferent arterioles. Increased erythropoietin results in increased hematocrit and hemoglobin, which decreases renal stress and sympathetic hyperactivity. Among others, these mechanisms have seen a reduction in all-cause mortality in patients with these conditions [8].

In recent years, there has been considerable evidence showing that SGLT-2 inhibitors can cause euglycemic diabetic ketoacidosis (EDKA) in patients who are undergoing surgery. This happens not only to those with diabetes but also to nondiabetic patients taking SGLT-2 inhibitors, although this is extremely rare [9,10].

In the case of EDKA, patients typically present with generalized malaise, fatigue, lethargy, loss of appetite, nausea, vomiting, abdominal pain, confusion, or shortness of breath. They may be tachycardic and develop a new oxygen requirement. Most importantly, patients have serum ketone levels above 3 mmol/L and increased anion gap metabolic acidosis. In contrast to diabetic ketoacidosis (DKA), hyperglycemia is absent in EDKA—as a result, there is usually no polyuria or polydipsia. EDKA should be considered a differential in postsurgical patients with these symptoms who are taking or have recently stopped SGLT-2 inhibitors.

Although the guidance for diagnosis and management of diabetic ketoacidosis is usually well documented, EDKA is relatively less known and indeed a unique phenomenon associated with the introduction and usage of SGLT-2 inhibitors. In recent years, an exponential rise has been noted in their prescriptions because of the added advantages these drugs offer, such as cardiovascular protection [11], renal protection [12], and metabolic benefits [13].

Two governmental reviews, one by the US Food and Drug Administration (FDA) and one by the European Medicines Agency, collectively identified 221 cases of DKA in patients undergoing planned surgery and who were taking SGLT-2 inhibitors. They both recognized that diagnosis and treatment of DKA was often missed in these patients because of a normal or near-normal serum glucose level [14,15]. In addition, a 2019 systematic review identified 42 cases of perioperative EDKA caused by SGLT-2 inhibitors [16].

Several specialist societies and some government health-care bodies have identified the need for definitive clinical guidelines on the perioperative management of these patients. However, it is unclear as to what the guidance is based upon and indeed if there is a discrepancy in the guidance across various health-care bodies. This can contribute to significant variations in clinical practice, such as whether the SGLT-2 inhibitors should be stopped preoperatively, if so for how long, and indeed what investigations and management plans should be in place for such patients to ensure optimal clinical outcomes. This article aims to collate and highlight the key points and inconsistencies in the existing guidelines.

## Material and methods

The PubMed database was searched from January 2010, the approximate date of their introduction to the market, to October 2024 with the MeSH terms “peri-operative”, “SGLT-2 inhibitors”, and “euglycemic DKA”. The search was supplemented through manual reading of references. Practice guidelines were the only publication type considered. Evidence used to formulate the guidelines was assessed and compared. The guidelines were compared in regard to the recommendations made.

## Results

Nine guidelines were identified and are listed in Table 2. They come from the UK, the United States, Australia, New Zealand, and Europe. Table 1 demonstrates how all but one guideline suggests withholding SGLT-2 inhibitors before surgery but vary in regard to when this should be done.

**Table 2:** Summary of recommendations.

Title	Author	Year	Recommended preoperative withholding time	Recommendation on ketone measurement	Recommended postoperative reinitiation time	Recommendation regarding emergency surgery
ADS-ANZCA Perioperative Diabetes and Hyperglycaemia Guidelines (Adults).	ADS and the ANZCA & Faculty of Pain Medicine.	2022	2 d. Suggests that SGLT-2i should NOT be stopped in patients without type 2 diabetes.	Hourly blood ketone measurement during the procedure, and 2-hourly measurement after the procedure until full oral intake is re-established.	2 d after major surgery, once ketones are normal and full oral intake is re-established.	Postprocedure, admit to a ward capable of managing EDKA and consult endocrinology and critical care.
SGLT2 Inhibitors—New Information on the Known Association Between SGLT2 Inhibitors and Diabetic Ketoacidosis in Surgical Patients.	European Medicines Agency.	2019	No specific recommendation, only that treatment with SGLT-2i should be “interrupted.”	No recommendation.	Once ketones are normal and full oral intake is re-established.	No recommendation.
Guideline for Perioperative Care for People With Diabetes Mellitus Undergoing Elective and Emergency Surgery.	Centre for Perioperative Care.	2021	1 d. “Longer” preoperative hold should be considered in those on a low-calorie diet and those that need preoperative bowel preparation.	Ketones should be measured daily throughout admission.	Once ketones are normal and full oral intake is re-established.	Ensure SGLT-2i are withheld.
Perioperative Management of the Surgical Patient With Diabetes.	Association of Anaesthetists of Great Britain and Northern Ireland.	2015	Withhold on the day of surgery. If having preoperative fasting, withhold from first day of fast. If having preoperative low-calorie diet, withhold from the first day of diet.	No recommendation.	No recommendation.	No recommendation.
Diabetes Care in the Hospital: Standards of Medical Care in Diabetes.	American Diabetes Association.	2024	3 d. 4 d for ertugliflozin.	No recommendation.	No recommendation.	No recommendation.
ESC Guidelines on Cardiovascular Assessment and Management of Patients Undergoing Noncardiac Surgery.	European Society of Cardiology.	2022	At least 3 d.	Measure ketones if there are signs of DKA.	No recommendation.	No recommendation.
Preoperative Cessation of SGLT2i.	American College of Cardiology.	2022	3-4 d.	No recommendation.	No recommendation.	No recommendation.
American Association of Clinical Endocrinologists and American College of Endocrinology Position Statement on the Association of SGLT-2 Inhibitors and Diabetic Ketoacidosis.	American Association of Clinical Endocrinologists and American College of Endocrinology.	2016	At least 24 hours.	Measure blood ketones and arterial pH if EDKA is suspected to confirm the diagnosis. Blood ketones should be measured instead of urinary ketones.	No recommendation.	Stop SGLT-2i immediately.
FDA Revises Labels of SGLT2 Inhibitors for Diabetes to Include Warnings About Too Much Acid in the Blood and Serious Urinary Tract Infections.	Food and Drug Administration.	2015	3 d. 4 d for ertugliflozin.	Consider monitoring ketones.	No recommendation.	No recommendation.

ADS, Australian Diabetes Society; ANZCA, Australian and New Zealand College of Anaesthetists; SGLT-2i, sodium-glucose linked transporter 2 inhibitors; ESC, European Society of Cardiology.

One guideline suggests withholding SGLT-2 inhibitors only on the day of surgery [17], 2 suggest withholding for 1 day before surgery [18,19], another suggests withholding for 2 days [20], and 4 guidelines suggest withholding for at least 3 days before surgery [15,[21], [22], [23]]. One guideline suggests withholding SGLT-2 inhibitors but makes no specific time recommendations [14]. Three guidelines suggest when to restart SGLT-2 inhibitors, and they advocate for restarting when serum ketones are normal and normal oral intake is re-established. Five guidelines advocate for regular monitoring of ketones. One of the guidelines [20] suggests that SGLT-2 inhibitors should not be withheld in people without type 2 diabetes, citing a lack of reports of EDKA in these patients. Three guidelines include a recommendation on emergency surgery; they all suggest stopping SGLT-2 inhibitors immediately, but only one advocates a clear strategy—to preemptively admit these patients to a ward capable of managing EDKA and with the input of endocrine and critical care specialists. There were no recommendations on how management of these patients should change with higher or lower doses of medication or longer duration of treatment before surgery.

Only the European Society of Cardiology guidelines used a grading system for their recommendations; its recommendation in regard to SGLT-2 inhibitors was ranked as class IIb, meaning that “weight of evidence/opinion is in favor of usefulness/efficacy”, and level C, meaning that it represents “consensus of opinion from the experts and/or small studies, retrospective studies, [and] registries.”

The recommendation for a 3-day preoperative withholding time for canagliflozin, dapagliflozin, and empagliflozin and a 4-day withholding time for ertugliflozin is seen twice: once in the original FDA guidelines and then in the American Diabetes Society guidelines, which bases its recommendation on the former. The evidence used to arrive at this recommendation is not specifically outlined by the FDA, but it seems to be based on the half-lives of these medications. The rest of the recommendations made in the guidelines do not have clear evidence behind them but seem to represent best clinical judgment and the literary consensus at the time at which they were written.

## Discussion

This review has identified significant variations in the recommendations made for perioperative management of patients who take SGLT-2 inhibitors. There is no consensus not only on the duration of withholding the medications but also on when to restart and what investigations to conduct while the patient is being monitored both intraoperatively and postoperatively.

The recommendations regarding the period of preoperative cessation of SGLT-2 inhibitors vary from 1 to 4 days. This represents a patient safety issue because a lack of understanding of the reasons behind a longer preoperative cessation period implies a lack of awareness of their potential to cause EDKA, which could mean that it is not considered a differential in an unwell surgical patient. The recommendations for reinitiation of SGLT-2 inhibitors, where they are made, all agree that this should only happen once serum ketones are within range and oral intake has returned to normal. This is a good thing; it shows awareness of the fact that the physiological stress caused by surgery must be at least partially abated before reintroducing the drug. The recommendations regarding the frequency of monitoring of serum ketones vary greatly in detail. The Australian and New Zealand College of Anaesthetists guidelines recommend hourly monitoring intraoperatively and 2-hourly monitoring postoperatively, until full oral intake is resumed. This is in contrast to the Centre for Preoperative Care guidelines, which recommend daily monitoring, and the other guidelines where ketone monitoring is mentioned, in which we are merely recommended to consider it. This is an important oversight; frequent monitoring would ensure that any developing cases of EDKA are caught and treated early.

DKA is defined as metabolic acidosis with increased serum ketone levels and increased serum glucose levels [24]. It normally occurs in patients with type 1 diabetes because of a lack of insulin, which forces the body to metabolize ketone bodies instead of glucose. EDKA is also characterized as increased anion-gap metabolic acidosis and increased serum ketones, but with a normal or near-normal serum glucose level [25].

**Figure 1:**
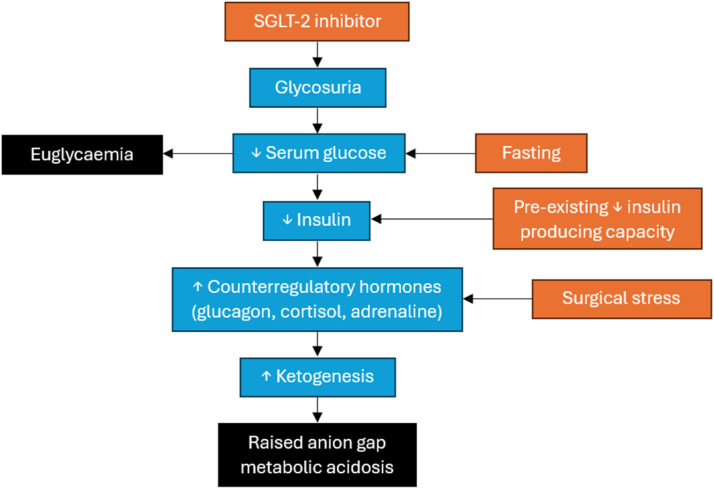
Mechanism of EDKA in surgical patients.

Surgery can provoke EDKA in patients taking SGLT-2 inhibitors for several reasons, as shown in Figure 1.

First, surgery represents a significant physiological stressor that increases serum concentrations of counter-regulatory hormones to insulin, thereby increasing insulin resistance and promoting ketogenesis.

Second, the reduction in serum glucose concentration caused by SGLT-2 inhibitors downregulates insulin and upregulates glucagon, which promotes the formation of ketone bodies and their use in respiration.

Third, surgical patients are typically fasted, which further exacerbates these effects when taking SGLT-2 inhibitors. Consequently, perioperative patients taking oral SGLT-2 inhibitors are at a significantly higher risk of developing DKA.

Finally, patients with a reduced capacity for insulin production for any reason, such as alcoholism, cystic fibrosis, or chronic pancreatitis to name a few, are at the highest risk of EDKA because of their impaired ability to counteract these combined challenges. All these factors can combine to result in failure to produce enough insulin to use glucose for respiration. This means that the body is forced to produce ketone bodies as an alternative energy source, which results in ketoacidosis despite a normal or near-normal serum glucose concentration.

Most SGLT-2 inhibitors have half-lives of 10-14 hours, with ertugliflozin having a half-life of 17 hours [26]. It takes around 5 half-lives for the serum concentration of a drug to be below clinically significant levels, meaning that SGLT-2 inhibitors can be present at clinically significant levels for up to 3 days (or 4 in the case of ertugliflozin) after they are ceased. In addition, there is evidence that SGLT-2 inhibitors may remain bound to receptors for longer than this and can exert their effects for up to 10 days [27]. One case series illustrates how EDKA can occur even when SGLT-2 inhibitors are ceased 3 days preoperatively and makes the point that duration of effect can be further lengthened in patients with renal impairment due to slower excretion [28]. These facts illustrate the importance of stopping SGLT-2 inhibitors for at least 3 days preoperatively and monitoring ketones regularly to ensure that any EDKA caused by a prolonged duration of action is diagnosed early.

The fact that the majority of existing guidelines do not seem to take the above physiology into account suggests that the dangers of SGLT-2 inhibitors in surgical patients have not been fully highlighted in the literature. Withholding SGLT-2 inhibitors for less than 3 days, as suggested by the majority of the guidelines, presents what could be seen as an unacceptable risk of EDKA in these patients, and failure to regularly monitor ketones further compounds the risk of a poor postoperative outcome. The fact that only 2 guidelines suggest when to restart SGLT-2 inhibitors is also concerning; restarting these drugs immediately after an operation fails to allow recovery from the physiological stress of fasting and surgery and could result in EDKA postoperatively. One guideline suggests that SGLT-2 inhibitors should not be withheld at all in patients without type 2 diabetes because of a lack of case reports describing this at the time of writing [20]; although rare, this is no longer true.

There is a paucity of case reports concerning EDKA in patients undergoing elective arthroplasty.

In light of all this, the following strategy could be considered reasonable for managing elective surgical patients, including elective arthroplasty patients, taking SGLT-2 inhibitors: first, instruct all patients to stop taking SGLT-2 inhibitors 4 days before they are due to arrive at the surgical center. Second, monitor serum ketones every 24 hours during the admission and the preoperative, intraoperative, and the immediate postoperative period. Serum ketones should be measured every postoperative day until their levels are within the normal range and oral intake has returned to normal—if the patient is being discharged the same day as the procedure, they should either be provided with a serum ketone meter to do this at home or report to the center to have this checked. Twenty-four-hour observation in hospital after the procedure is probably not warranted for this reason alone; instead, patients should monitor serum ketones as previously mentioned and be advised to report to the hospital if they are feeling any of the following symptoms: generalized malaise, fatigue, lethargy, loss of appetite, nausea, vomiting, abdominal pain, confusion, or shortness of breath. Serum ketones are more accurate than urinary ketones and can be measured instantly. Third, only restart SGLT-2 inhibitors once serum ketones are within range and oral intake has returned to normal.

Withholding SGLT-2 inhibitors may result in increased blood sugar. While undesirable, this is better than running the risk of EDKA. However, in patients with poorly controlled diabetes, starting sliding scale insulin should be considered on admission. In patients undergoing unplanned surgery, it seems prudent to postoperatively admit to a ward capable of managing EDKA, with the advice of appropriate specialists.

## Conclusions

Use of SGLT-2 inhibitors is on the rise. Many patients taking these drugs will need surgery at some point; as such, it is crucial to be aware of the physiological effects of SGLT-2 inhibitors and how the stress of surgery can compound the effects to cause EDKA.

It is clear that although there have been advances in the understanding of this problem in recent years, more needs to be done to disseminate this information. In essence, physicians and surgeons should be aware that SGLT-2 inhibitors should be withheld for at least 3 days preoperatively, that serum ketones should be monitored regularly, that SGLT-2 inhibitors should only be restarted once both serum ketone levels and oral intake are back to normal, and that these measures should be undertaken regardless of whether the patient has diabetes.

## Conflicts of interest

Professor Hemant Pandit is supported in part by the National Institute for Health and Care Research Leeds Biomedical Research Centre (NIHR203331). The views expressed are those of the authors and not necessarily those of the NHS, the National Institute for Health and Care Research or the Department of Health and Social Care. The other authors declare no potential conflicts of interest.

For full disclosure statements refer to https://doi.org/10.1016/j.artd.2025.101840.

## CRediT authorship contribution statement

**James H.J. Selbie:** Writing – original draft. **Shuhei Hiyama:** Writing – review & editing. **Hemant Pandit:** Writing – review & editing, Conceptualization.

## Appendix A Supplementary data

Conflict of Interest Statement for Pandit

Conflict of Interest Statement for Selbie

Conflict of Interest Statement for Hiyama
