# Supplementary material for: Euglycemic Diabetic Ketoacidosis and Its Prevention in Elective Surgical Patients Taking Sodium-Glucose Linked Transporter 2 Inhibitors: An International Perspective
Source: Arthroplast Today. 2025 Sep 16;35:101840. doi: 10.1016/j.artd.2025.101840 (PMC12464960; doi:10.1016/j.artd.2025.101840)
Supplement: Conflict of Interest Statement for Pandit [file mmc1.docx]

# INDIVIDUAL CONFLICT OF INTEREST STATEMENT

***American Association of Hip and Knee Surgeons***

(Adopted from the American Academy of Orthopaedic Surgeons disclosure statement)

The following form **must be filled out completely and submitted by each author (example, 6 authors, 6 forms).**

**All items require a response. If there is no relevant disclosure for a given item, enter "*None*.”**

Euglycaemic diabetic ketoacidosis and its prevention in elective surgical patients taking SGLT-2 inhibitors: an international perspective

**Manuscript Title**

1. Royalties from a company or supplier (The following conflicts were disclosed) None

2. Speakers bureau/paid presentations for a company or supplier (The following conflicts were disclosed) None

3A. Paid employee for a company or supplier (The following conflicts were disclosed) None

3B. Paid consultant for a company or supplier (The following conflicts were disclosed) Medacta International, Zimmer Biomet, Depuy Synthes, Invibio, MAT Ortho, Microport International, Allay Therapeutics, Paradigm Pharmaceuticals

3C. Unpaid consultants for a company or supplier (The following conflicts were disclosed) None

4. Stock or stock options in a company or supplier (The following conflicts were disclosed) Allay Therapeutics

5. Research support from a company or supplier as a Principal Investigator (The following conflicts were disclosed) Medacta International, Zimmer Biomet, Depuy Synthes, Invibio, Allay Therapeutics, Paradigm Pharmaceuticals

6. Other financial or material support from a company or supplier (The following conflicts were disclosed) None

7. Royalties, financial or material support from publishers (The following conflicts were disclosed) None

8. Medical/Orthopaedic publications editorial/governing board (The following conflicts were disclosed) None

9. Board member/committee appointments for a society (The following conflicts were disclosed) None

**Each author must sign AND print or type his/her name, date and submit a separate form**

In addition, one BLINDED Conflict of Interest form (no author names used) should be submitted per manuscript with all author disclosures.

Hemant Pandit
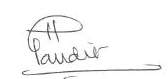
 10^th^ Jan 2025

Author Name (Print or Type) Author Signature Date
