# Supplementary material for: Euglycemic Diabetic Ketoacidosis and Its Prevention in Elective Surgical Patients Taking Sodium-Glucose Linked Transporter 2 Inhibitors: An International Perspective
Source: Arthroplast Today. 2025 Sep 16;35:101840. doi: 10.1016/j.artd.2025.101840 (PMC12464960; doi:10.1016/j.artd.2025.101840)
Supplement: Conflict of Interest Statement for Hiyama [file mmc3.docx]

# INDIVIDUAL CONFLICT OF INTEREST STATEMENT

***American Association of Hip and Knee Surgeons***

(Adopted from the American Academy of Orthopaedic Surgeons disclosure statement)

The following form **must be filled out completely and submitted by each author (example, 6 authors, 6 forms).**

**All items require a response. If there is no relevant disclosure for a given item, enter "*None*.”**

**Manuscript Title:** Euglycaemic diabetic ketoacidosis and its prevention in elective surgical patients taking SGLT-2 inhibitors: an international perspective.

1. Royalties from a company or supplier (The following conflicts were disclosed) None

2. Speakers bureau/paid presentations for a company or supplier (The following conflicts were disclosed) None

3A. Paid employee for a company or supplier (The following conflicts were disclosed) None

3B. Paid consultant for a company or supplier (The following conflicts were disclosed) None

3C. Unpaid consultants for a company or supplier (The following conflicts were disclosed) None

4. Stock or stock options in a company or supplier (The following conflicts were disclosed) None

5. Research support from a company or supplier as a Principal Investigator (The following conflicts were disclosed) None

6. Other financial or material support from a company or supplier (The following conflicts were disclosed) None

7. Royalties, financial or material support from publishers (The following conflicts were disclosed) None

8. Medical/Orthopaedic publications editorial/governing board (The following conflicts were disclosed) None

9. Board member/committee appointments for a society (The following conflicts were disclosed) None

**Each author must sign AND print or type his/her name, date and submit a separate form**

In addition, one BLINDED Conflict of Interest form (no author names used) should be submitted per manuscript with all author disclosures.

Shuhei Hiyama
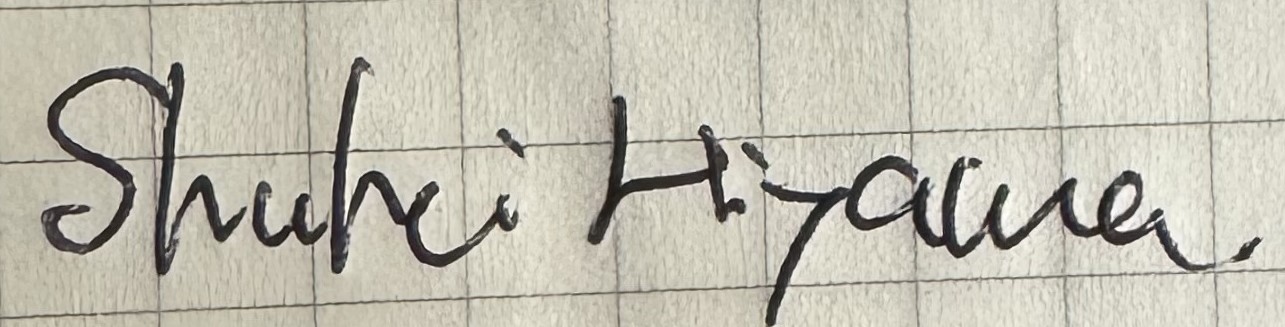
 09 Jan. 2025

Author Name (Print or Type) Author Signature Date
